# Supplementary material for: Heat adaptation of phage T7 under an extended genetic code
Source: Virus Evol. 2021 Dec 1;7(2):veab100. doi: 10.1093/ve/veab100 (PMC8923235; doi:10.1093/ve/veab100)
Supplement: veab100_Supp [file veab100_supp.zip › Sequencing_Analysis.zip!/Sequencing_Analysis/A3/evidence/RA_44.html]

BRESEQ :: Evidence


| Read alignment evidence... | | | | | | | | | | | |
| --- | --- | --- | --- | --- | --- | --- | --- | --- | --- | --- | --- |
|  | seq id | position | | ref | new | freq | score (cons/poly) | reads | annotation | genes | product |
| \* | NC\_001604.1 | 33,023 | 0 | A | G | 38.2% | 1351.7 / inf | 2086 | intergenic (–/–) | *–/–* | –/– |
| Reads supporting (aligned to +/- strand):  **ref** base A (658/620);  **new** base G (398/393);  **total** (1066/1020) | | | | | | | | | | | |
| Fisher's exact test for biased strand distribution *p*-value = 6.19e-01 | | | | | | | | | | | |
| Kolmogorov-Smirnov test that lower quality scores support variant *p*-value = 6.07e-01 | | | | | | | | | | | |

Only 100 of 2092 total aligned reads displayed.  

`ATGAGATGCTTAAGGAATTACACGGGGTGAAGGAAGTAACACCAGAGATGGTAGAGAAGTACGCTATGGATAAGGCTTATGGTATCTCCCACTCAGACCAGTTCACCAACAGTTCCATAATAGAAGAGAACATTGAGGGCTTAGTAGGTATCGAGAATAACTCATTCCTTGAGGCACGTAACTTGTTTGATTCGGACCTATCCATCACTATGCCAGACGGACAGCAATTCTCAGTGAATGACCTAAGGGACTTCGATATGTTCCGCATCATGCCAGCGTATGACCGCCGTGTCAATGGTGACATCGCCATCATGGGGTCTACTGGTAAAACCACTAAGGAACTTAAGGATGAGATTTTGGCTCTCAAAGCGAAAGCTGAGGGAGACGGTAAGAAGACTGGCGAGGTACATGCTTTAATGGATACCGTTAAGATTCTTACTGGTCGTGCTAGACGCAATCAGGACACTGTGTGGGAAACCTCACTGCGTGCCATCAATGACCTAGGGTTCTTCGCTAAGAACGCCTACATGGGTGCTCAGAACATTACGGAGATTGCTGGGATGATTGTCACTGGTAACGTTCGTGCTCTAGGGCATGGTA  >  NC_001604.1/32723‑33322`  

                                                                                                                                                                                                                                                                                                            `|`                                                                                                                                                                                                                                                                                                             
        `cTTAAGGAATTACACGGGGTGAAGGAAGTAACACCAGAGATGGTAGAGAAGTACGCTATGGATAAGGCTTATGGTATCTCCCACTCAGACCAGTTCACCAACAGTTCCATAATAGAAGAGAACATTGAGGGCTTAGTAGGTATCGAGAATAACTCATTCCTTGAGGCACGTAACTTTTTTGATTCGGACCAATCCATCACTATGCCAGACGGACAGCAATTCTCAGTGAATGACCTAAGGGACTTCGATATGTTCCGCATCATGCCAGCGTATGACCGCCGTGTCAATGTTTACCTCGccc                                                                                                                                                                                                                                                                                                     >  1:318527/1‑300 (MQ=255)`  
                        `gggTGAAGGAAGTAACACCAGAGATGGTAGAGAAGTACGCTATGGATAAGGCTTATGGTATCTCCCACTCAGACCAGTTCACCAACAGTTCCATAATAGAAGAGAACATTGAGGGCTTAGTAGGTATCGAGAATAACTCATTCCTTGAGGCACGTAACTTGTTTGATTCGGACCTATCCATCACTATGCCAGACGGACAGCAATTCTCAGTGAATGACCTAAGGGACTTCGATATGTTCCGCATCATGCCAGCGTATGACCGCCGTGTCAATGGTGACATCGCCATCATGGGGTCTACTgt                                                                                                                                                                                                                                                                                     >  1:264637/1‑300 (MQ=255)`  
                        `gggTGAAGGAAGTAACACCAGAGATGGTAGAGAAGTACGCTATGGATAAGGCTTATGGTATCTCCCACTCAGAACAGTTCACCAACAGTTCCATAATAGAAGAGAACATTGAGGGCTTAGTAGGTATCGAGAATAACTCATTCCTTGAGGCACGTAACTTGTTTGATTCGGACCTATCCATCACTATGCCAGACGGACAGCAATTCTCAGTGAATGACCTAAGGGACTTCGATATGTTCCGCATCATGCAAGCGTATGACCGCCGTGTCAATGGTGACATCGCCATAATGGGGTCTACTgg                                                                                                                                                                                                                                                                                     >  1:54677/1‑301 (MQ=255)`  
                         `ggTGAAGGAAGTAACACCAGAGATGGTAGAGAAGTACGCTATGGATAAGGCTTATGGTATCTCCCACTCAGACCAGTTCACCAACAGTTCCATAATAGAAGAGAACATTGAGGGCTTAGTAGGTATCGAGAATAACTCATTCCTTGAGGCACGTAACTTGTTTGATTCGGACCTATCCATCACTATGCCAGACGGACAGCAATTCTCAGTGAATGACCTAAGGGACTTCGATATGTTCCGCATCATGCCAGCGTATTACCGCCGTGTCAATGGTGGCATCGCCATCATGGGGTCTACTGGt                                                                                                                                                                                                                                                                                    <  1:288550/301‑1 (MQ=255)`  
                          `gTGCAGGAAGTAACACCAGAGATTGTAGAGAAGTACGCTATGGATAAGGCTTATGGTATCTCCCACTCAGACCAGTTCACCAACAGTTCCATAATAGAAGAGAACATTGAGGGCTTAGTAGGTATCGAGAATAACTCATTCCTTGAGGCACGTAACTTGTTTGATTCGGACCTATCCATCACTATGCCATACGGACAGCAATTCTCAGTGAATGACCTAAGGGACTTCGATATGTTCCGCATCATGCCAGCGTATGACCGCCGTGTCAATGGTGGCATCGCCATCATGGGGTcctgtctct                                                                                                                                                                                                                                                                                   >  1:251992/1‑293 (MQ=255)`  
                          `gTGAAGTAAGTAACACAAGAGATGGTAGAGAAGTACGCTATGGATAAGGCTTATGGTATCTCCCACTCAGACCAGTTCACCAACAGTTCCATAATAGAAGAGAACATTGAGGGCTTAGTAGGTATCGAGAATAACTCATTCCTTGAGGCACGTAACTTGTTTGATTCGGACCTATCCATCACTATGCCAGACGGACAGCAATTCTCAGTGAATGACCTAAGGGACTTCGATATGTTCCGCATCATGCCAGCGTATGACCGCCGTGTCAATGGTGGCATCGCCATCATGGGGTCTACTGGTa                                                                                                                                                                                                                                                                                   <  1:335611/301‑1 (MQ=255)`  
                            `gaaggaagTAACACCAGAGATGGTAGAGAAGTACGCTATGGATAAGGCTTATGGTATCTCCCACTCAGACCAGTTCACCAACAGTTCCATAATAGAAGAGAACATTGAGGGCTTAGTAGGTATCGAGAATAACTCATTCCTTGAGGCACGTAACTTGTTTGATTCGGACCTATCCATCACTATGCCAGACGGACAGCAATTCTCAGTGAATGACCTAAGGGACTTCGATATGTTCCGCATCATGCCAGCGTATGACCGCCGTGTCAATGGTGGCATCGCCATCATGGGGTCTAcctgtctc                                                                                                                                                                                                                                                                                 >  1:97047/1‑294 (MQ=255)`  
                               `ggaagTAACACCAGAGATGGTAGAGAAGTACGCTATGGATAAGGCTTATGGTATCTCCCACTCAGACCAGTTCACCAACAGTTCCATAATAGAAGAGAACATTGAGGGCTTAGTAGGTATCGAGAATAACTCATTCCTTGAGGCACGTAACTTGTTTGATTCGGACCTATCCATCACTATGCCAGACGGACAGCAATTCTCAGTGAATGACCTAAGGGACTTCGATATGTTCCGCATCATGCCAGCGTATGACCGCCGTGTCAATGGTTACATCGCCATTATGGGGTCTACTGGTAAAAcc                                                                                                                                                                                                                                                                              >  1:303947/1‑301 (MQ=255)`  
                                 `aagTAACACCAGAGATGGTAGAGAAGTACGCTATGGATAAGGCTTATGGTATCTCCCACTCAGACCAGTTCACCAACAGTTCCATAATAGAAGAGAACATTGAGGGCTTAGTAGGTATCGAGAATAACTCATTCCTTGAGGCACGTAACTTGTTTGATTCGGACCTATCCATCACTATGCCAGACGGACAGCAATTCTCAGTGAATGACCTAAGGGACTTCGATATGTTCCGCATCATGCCAGCGTATGACCGCCGTGTCAATGGTGACATAGCCATCATGGGGTCtctgtctcttataca                                                                                                                                                                                                                                                                            >  1:19519/1‑287 (MQ=255)`  
                                         `aagagacaggTAGAGAAGTACGCTATGGATAAGGCTTATGGTATCTCCCACTCAGACCAGTGCACCAACAGTTCCATAATAGAAGAGAACATTGAGTGCTTAGTAGGTATCGAGAATAACTCATTCCTTGAGGCACGTAACTTGTTTGATTCGGACCTATCCATCACTATGCCAGACGGACAGCAATTCTCAGTGAATGACCTAAGGGACTTCGATATGTTCCGCATCATGCCAGCGTATGACCGCCGTGTCAATGGTGGCATCGCCATCATGGGGTCTACTTGTAAAACCACTAAGGAAc                                                                                                                                                                                                                                                                    <  1:331929/293‑1 (MQ=255)`  
                                           `agagATGGTAGAGAAGTACGCTATGGATAAGGCTTATGGTATCTCCCACTCAGAACAGTTCACCAACAGTTCCATAATAGAAGAGAACATTGAGGGCTTAGTAGGTATCGAGAATAACTCATTACTTGAGGCAAGTAACTTGTTTGATTCGGAAATATCCATCACTATGCAAGACGGACAGCAATTATAAGTGAATGACCTAAGGGACTTCGATATGTTCCGCATAATGCCAGCGTATGACCGCAGTGTCAATGGTGACATAGCGATCATGGGGTATCCTGGTACAACCACTAAggctgtc                                                                                                                                                                                                                                                                  >  1:149424/1‑296 (MQ=255)`  
                                             `tgggctcggagatgtgtataagagacagggCTTATGGTATCTCCCACTCAGACCAGTTCACCAACAGTTCCATAATAGAAGAGAACATTGAGGGCTTAGTAGGTATCGAGAATAACTCATTCCTTGAGGCACGTAACTTGTTTGATTCGGACCTATCCATCACTATGCCAGACGGACAGCAATTCTCAGTGAATGACCTAAGGGACTTCGATATGTTCCGCATCATGCCAGCGTATGACCGCCGTGTCAATGGTGGCATCGCCATCATGGGGTCTACTGGTAAAACCACTAAGGAACTTaa                                                                                                                                                                                                                                                                <  1:341459/273‑1 (MQ=255)`  
                                               `aTGGTAGAGAAGTACGCTATGGATAAGGCTTATGGTATCTCCCACTCAGACCAGTTCACCAACAGTTCCATAATAGAAGAGAACATTGAGGGCTTAGTAGTTATCGAGAATAACTCATTCCTTGAGGCACGTAACTTGTTTGATTCGGACCTATCCATCACTATGCCAGACGGACAGCAATTCTCAGTGAATGACCTAAGGGACTTCGATATGTTCCGCATCATGCCAGCGTCTGACCGCCGTGTCAATGGTGACATCGCCAACATGGGGTCTACTGGTAAAACCACTAAGGAACTTAAgg                                                                                                                                                                                                                                                              >  1:238625/1‑301 (MQ=255)`  
                                                `tGGTAGAAAAGTACGCTATGGATAATGCTTATGGTATCTCCCACTCAGACCAGTTCACCAACAGTTCCATAATAGAAGAGAACATTGAGGGCTTAGTAGGTATCGAGAATAACTCATTCCTTGAGGCACGTAACTTGTTTGATTCGGACCTATCCATCACTATGCCAGACGGACAGCAATTCTCAGTGAATGACCTAAGGGCCTTCGATATGTTCCGCATCATGCCAGCGTATGACCGCCGTGTCAATGGTGACATCGCCATCATGGGGTCTACTGGTAAAACCACTAAGGAACTTAATGa                                                                                                                                                                                                                                                             <  1:44489/301‑1 (MQ=255)`  
                                                          `gTACGCTATGGATAAGGCTTATGGTATCTCCCACTCAGACCAGTTCACCAACAGTTCCATAATAGAAGAGAACATTGAGGGCTTAGTAGGTATCGAGAATAACTCATTCCTTGAGGCACGTAACTTGTTTGATTCGGACCTATCCATCACTATGCCAGACGGACAGCAATTCTCAGTGAATGACCTAAGGGACTTCGATATGTTCCGCATCATGCCAGCGTATGACCGCCGTGTCAATGGTGACATCGCCATCATGGGGTCTACTGGTAAAACCACTAAGGAACTTAAGGATGAGATTTTg                                                                                                                                                                                                                                                   >  1:48252/1‑301 (MQ=255)`  
                                                                 `aTGGATAAGGCTTATGGTATCTCCCACTCAGACCAGTTCACCAACAGTTCCATAATAGAAGAGAACATTGAGGGCTTAGTAGGTATCGAGAATAACTCATTCCTTGAGGCACGTAACTTGTTTGATTCGGACCTATCCATCACTATGCCAGACGGACAGCAATTCTCAGTGAATGACCTAAGGGACTTCGATATGTTCCGCATCATGCCAGCGTATGACCGCCGTGTCAATGGTGACATCGCCATCATGGGGTCTCCTGGTAAAACCACTAATGAACTTAAGGATgctgtctcttataacc                                                                                                                                                                                                                                            >  1:18439/1‑286 (MQ=255)`  
                                                                   `ggATAAGGCTTATGGTATCTCCCACTCAGACCAGTTCACCAACAGTTCCATAATAGACGAGAACATTGAGGGCTTAGTAGGTATCGAGATTTACTCTTCCCTTGATGCACGTAACTTGTTTGATTCGGACCTCTCCATCACTTTGCTAGACGGACAGCACTTCTCAGAGACTTACCTACGGGCCTTCGATATGTTCCGCATCATGCCATAGTCTGACCGCCGTGTCTATTGTGACATCGCCGCCAGTGTGTCTGCCGGTTAAACCACTCAGGAGCTTAATGCTGAGATTGTGTcttataca                                                                                                                                                                                                                                          >  1:208845/1‑295 (MQ=255)`  
                                                                         `ggCTTATGGTATCTCCCACTCAGACCAGTTCACCAACAGTTCCATAATAGAAGAGAACATTGAGGGCTTAGTAGTTATCGAGAATAACTCATTCCTTGAGGCACGTAACTTGTTTGATTCGGACCTATCCATCACTATGCCAGACGGACAGCAATTCTCAGTGAATGACCTAAGGGACTTCGATATGTTCCGCATCATGCCAGCGTATGACCGCCGTGTCAATGGTGACATCGCCATCATGGGGTCTACTGGTAAAACCACTAAGGAACTTAAGGATGAGATTTTGGCTCTCAAAGCaaaa                                                                                                                                                                                                                                    >  1:266947/1‑301 (MQ=255)`  
                                                                         `ggCTTATGGTATCTCCCACTCAGACCAGTTCACCAACAGTTCCATAATAGAAGAGAACATTGAGGGCTTAGTAGGTATCGAGAATAACTCATTCCTTGAGGCACGTAACTTGTTTGATTCGGACCTATCCATCACTATGCCAGACGGACAGCAATTCTCAGTGAATGACCTAAGGGACTTCGATATGTTCCGCATCATGCCAGCGTATGACCGCCGTGTCAATGGTGACATCGCCATCATGGGGTCTACTGGTAAAACCACTAAGGAACTTAAGGATGAGATTTTGGCTCTCAAAGCGaaa                                                                                                                                                                                                                                    >  1:143300/1‑301 (MQ=255)`  
                                                                                     `ctcCCACTCAGACCAGTTCACCAACAGTTCCATAATAGAAGAGAACATTGAGGGCTTAGTAGGTATCGAGAATAACTCATTCCTTGAGGCACGTAACTTGTTTGATTCGGACCTATCCATCACTATGCCAGACGGACAGCAATTCTCAGTGAATGACCTAAGGGACTTCGATATGTTCCGCATCATGCCAGCGTATGACCGCCGTGTCAATGGTGACATCGCCATCATGGGGTCTACTGGTAAAACCACTAAGGAACTTAATGATGAGATTTTGGCTCTCAAAGCGAAAGCTGAGGGAGAc                                                                                                                                                                                                                        >  1:212030/1‑301 (MQ=255)`  
                                                                                          `aCTCAGACCAGTTCACCAACAGTTCCATAATAGAAGAGAACATTGAGGGCTTAGTAGGTATCGAGAATAACTCATTCCTTGAGGCACGTAACTTGTTTGATTCGGACCTATCCATCACTATGCCAGACGGACAGCAATTCTCAGTGAATGACCTAAGGGACTTCGATATGTTCCGCATCATGCCAGCGTATGACCGCCGTGTCAATGGTGGCATCGCCATCATGGGGTCTACTGGTAAAACCACTAAGGAACTTAAGGATGAGATTTTGGCTTTCAAAGCGAAAGATGAGGGAGACGGTaa                                                                                                                                                                                                                   >  1:219382/1‑301 (MQ=255)`  
                                                                                          `aCTCAGACCAGTTCACCAACAGTTCCATAATAGAAGAGAACATTGAGGGCTTAGTAGGTATCGAGAATAACTCATTCCTTGAGGCACGTAACTTGTTTGATTCGGACCTATCCATCACTATGCCAGACGGACAGCAATTCTCAGTGAATGACCTAAGGGACTTCGATATGTTCCGCATCATGCCAGCGTATGACCGCCATGTCAATGGTGACATCGCCATCATGGGGTCTACTGGTAAAACCACTAAGGAACTTAAGGATGAGATTTTGGCTCTCAAAGCGAAAGCTGAGGGAGAAGGTaa                                                                                                                                                                                                                   >  1:245503/1‑301 (MQ=255)`  
                                                                                                `gagacagcACCAACAGTTCCATAATAGAAGAGAACATTGAGGGCTTAGTAGGTATCGAGAATAACTCATTCCTTGAGGCACGTAACTTGTTTGATTCGGACCTATCCATCACTATGCCAGACGGACAGCAATTCTCAGTGAATGACCTAAGGGACTTCGATATGTTCCGCATCATGCCAGCGTATGACCGCCGTGTCAATGGTGGCATCGCCATCATGGGGTCTACTGGTAAAACCACTAAGGAACTTAAGGATGAGATTTTGGCTCTCAAAGCGAAAGCTGAGGGAGACGGTAAGAAGAc                                                                                                                                                                                                             <  1:280000/294‑1 (MQ=255)`  
                                                                                                    `gTTCACCAACAGTTCCATAATAGAAGAGAACATTGAGGGCTTAGTAGGTATCGAGAATAACTCATTCCTTGAGGCACGTAACTTGTTTGATTCGGAACTATCCATCACTATGCCAGACGGACAGCAATTCTCAGTGAATGACCTAAGGGACTTCGATATGTTCCGCATCATGCCAGCGTATGACCGCCGTGTCAATGGTGACATCGCCATCATGGGGTCTACTGGTAAAACCACTAAGGAACTTAAGGATGAGATTTTGCCTCTCAAAGCGAAAGCTGAAGGAGACGGTAAGAAGACTggg                                                                                                                                                                                                         >  1:282047/1‑300 (MQ=255)`  
                                                                                                     `ggagatgtttataagagacaggaagaGAACATTGAGGGCTTAGTAGATATCGAGAATAACTCATTCCTTGAGGCACGTAACTTGTTTGATTCGGACCTATCCATCACTATGCCAGACGGACAGCAATTCTCAGTGAATGACCTAAGGGACTTCGATATGTTCCGCATCATGCCAGCGTATGACCGCCGTGTCAATGGTGACATCGCCATCATGGGGTCTACTGGTAAAACCACTAAGGAACTTAAGGATGAGATTTTGGCTCTCAAAGCGAAAGCTGAGGGAGACGGTAAGAAGACTGGCg                                                                                                                                                                                                        <  1:177117/280‑1 (MQ=255)`  
                                                                                                       `cACCAACAGTTCCATAATAGAAGAGAACATTTAGGGCTTAGTAGGTATCGAGAATAACTCATTCCTTGAGGCACGTAACTTGTTTGATTCGGACCTATCCATCACTATGCCAGACGGACAGCAATCCTCAGTGAATGACCTAAGGGACTTCGATATGTTCCGCATCATGCCAGCGTATGACCGCCGTGTCAATGGTGACATCGCCATCATGGGGTCTACTGGTAAAACTACTCAGGAACTTAAGGATGAGATTTTGGCTCTCAAAGCGAAAGCTGAGGGAGACGGTAAGAAGACTGGCGAg                                                                                                                                                                                                      >  1:153442/1‑301 (MQ=255)`  
                                                                                                       `cACCAACAGTTCCATAATAGAAGAGAACATTGAGGGCTTAGTAGGTATCGAGAATAACTCATTCCTTGAGGCACGTAACTTGTTTGATTCGGACCTATCCATCACTATGCCAGACGGACAGCAATTCTCAGTGAATGACCTAAGGGACTTCGATATGTTCCGCATCATACCAGCGTATGACCGCCGTGTCAATGGTGGCATCGCCATCATGGGGTCTACTGGTAAAACCACTAAGTAACTTAAGGATGAGATTTTGGCTCTCAAAGCGAAAGCTGAGGGAGACGGTAAGAAGACTGGctgt                                                                                                                                                                                                      >  1:258265/1‑298 (MQ=255)`  
                                                                                                           `ctcggagatgtgtataagagacagaTTGAGGGCTTAGTAGGTATCGAGAATAACTCATTCCTTGAGGCACGTAACTTGTTTGATTCGGACCTATCCATCACTATGCCAGACGGACAGCAATTCTCAGTGAATGACCTAAGGGACTTCGATATGTTCCGCATCATGCCAGCGTATGACCGCCGTGTCAATGGTGACATCGCCATCATGGGGTCTACTGGTAAAACCACTAAGGAACTTAAGGATGAGATTTTGGCTCTCAAAGCGAAAGCTGAGGGAGACGGTAAGAAGACTGGCGAGGTAc                                                                                                                                                                                                  <  1:158373/277‑1 (MQ=255)`  
                                                                                                               `gTTCCATAATAGAAGAGAACATTGAGGGCTTAGTAGGTATCGAGAATAACTCATTCCTTGAGGCACGTAACTTGTTTGATTCGGACCTATCCATCACTATGCCAGACGGACAGCAATTCTCAGTGAATGACCTAAGGGACTTCGATATGTTCCGCATCATGCCAGCGTATGACCGCCGTGTCAATGGTGACATCGCCATCATGGGGTCTACTGGTAAAACCACTAAGGAACTTAATGATGAGATTTTGGCTCTCAAAGCGAAAGCTGAGGGAGACGGTAAGAAGACTGGCGAGGTACATGc                                                                                                                                                                                              <  1:5963/301‑1 (MQ=255)`  
                                                                                                               `gTTCCATAATAGAAGAGAACATTGAGGGCTTAGTAGGTATCAAGAATAACTCATTCCTTGAGGCACGTAACTTGTTTGATTCGGACCTATCCATCACTATGCCAGACGGACAGCAATTCTCAGTGAATGACCTAAGGGACTTCGATATGTTCCGCATCATGCCAGCGTATGACCGCCGTGTCAATGGTGACATCGCCATCATGTGGTCTACTGGTAAAACCACTAAGGAACTTAAGGATGAGATTTTGGCTCTCAACGCGAAAGCTGAGGGAGAcctgtctcttatacacatctccgagcc                                                                                                                                                                                              >  1:24230/1‑275 (MQ=255)`  
                                                                                                               `aaggagatgtgtataagatacaggAGGGCTTAGTACGTATGTAGAATACATCAGTCCTTGAGGGACGTAACTTGTTTAATTCGGACCAATCCATCACTATGACAGATGGACAGCAATTCTCAGTGAATGTCATAAGGTACTTCGATATATTCCGCATCATGCCAGCGAATGACCGCCGTGTCAATGGTGGCATCGCCATCAAGGGGTCTACTCGTAAGACCACTAAGGAACTTAAGGATGAGATTTTGGCTCTCAAAGCGAAAGCTGAGGGAGACGGTAAGAAGACTGGCGAGGTACATGc                                                                                                                                                                                              <  1:22184/278‑1 (MQ=255)`  
                                                                                                                `atgtgtataagagacagaCATTGAGGGCTTAGTAGGTATCGAGAATAACTCATTCCTTGAGGCACGTAACTTGTTTGATTCGGACCTATCCATCACTATGCCAGACGGACAGCAATTCTCAGTGAATGACCTAAGGGACTTCGATATGTTCCGCATCATGCCAGCGTATGACCGCCGTGTCAATGGTGACATCGCCATCATGGGGTCTACTGGTAAAACCACTAAGGAACTTAAGGATGAGATTTTGGCTCTCAAAGCGAAAGCTGAGGGAGACGGTAAGAAGACTGGCGAGGTACAAGCt                                                                                                                                                                                             <  1:322220/284‑1 (MQ=255)`  
                                                                                                                     `taataGAAGAGAACATTGAGGGCTTAGTAGGTATCGAGAATAACTCATTCCTTGAGGCACGTAACTTGTTTGATTCGGACCTATCCATCACTATGCCAGACGGACAGCAATTCTCAGTGAATGACCTAAGGGACTTCGATATGTTCCGCATCATGCCAGCGTATGACCGCCGTGTCAATGGTGGCATCGCCATCATGGGGTCTACTGGTAAAACCACTAAGGAACTTAAGGATGAGATTTTGGCTCTCAAAGCGAAAGCTGAGGGAGACGGTAAGAAGACTGGCGAGGTACATGCTTTact                                                                                                                                                                                        >  1:230024/1‑299 (MQ=255)`  
                                                                                                                     `taataGAAGAGAACATTGAGGGCTTAGTAGGTATCGAGAATAACTCATTCCTTGAGGCACGTAACTTGTTTGATTCGGACCTATCCATCACTATGCCAGACGGACAGCAATTCTCAGTGAATGACCTAAGGGACTTCGATATGTTCCGCATCATGCCAGCGTATGACCGCCGTGTCAATGGTGACATCGCCATCATGGGGTCTACTGGTAAAACCACTAAGGAACTTAAGGATGAGATTTTGGCTCTCACAGCGAAAGCTGAGGGAGACGGTAAGAAGAATGGCGCGGTAAATGCTTTAAt                                                                                                                                                                                        >  1:66858/1‑301 (MQ=255)`  
                                                                                                                     `taataGAAGAGAACATTGAGGGCTTAGTAGGTATCGAGAATAACTCATTCCTTGAGGCACGTAACTTGTTTGATTCGGACCTATCCATCACTATGCCAGACGGACAGCAATTCTCAGTGAATGACCTAAGGGACTTCGATATGTTCCGCATCATGCCAGCGTATGACCGCCGTGTCAATGGTGACATCGCCATCATGGGGTCTACTGGTAAAACCACTAAGGAACTTAAGGATGAGATTTTGGCTCTCAAAGCGAAATCTGAGTGAGACGGTAAGAAGACTGGCGAGGTACATGCTTTAAt                                                                                                                                                                                        >  1:285600/1‑301 (MQ=255)`  
                                                                                                                      `acaggaagaGAACATTGAGGGCTTAGTAGGTATCGAGAATAACTCATTCCTTGAGGCACGTAACTTGTTTGATTCGGACCTATCCATCACTATGCCAGACGGACAGCAATTCTCAGTGAATGACCTAAGGGACTTCGATATGTTCCGCATCATGCCAGCGTATGACCGCCGTGTCAATGGTGACATCGCCATCATGGGGTCTACTGGTAAAACCACTAAGGAACTTAAGGATGAGATTTTGGCTCTCAAAGCGAAAGCTGAGGGAGACGGTAAGAAGACTGGCGAGGTACATGCTTTAATg                                                                                                                                                                                       <  1:137325/297‑1 (MQ=255)`  
                                                                                                                       `ataGAAGAGAACATTGAGGGCTTAGTAGGTATCGAGAATAACTCATTCCTTGAGGCACGTAACGTGTTTGATTCGGACCTATCCATCACTATGCCAGACGGACAGCAATGCTCAGTGAATGACCTAAGGGACTTCGATATGTTCCGCATCATGCCAGCGTATGACCGCCGTGTCAATGGTGGCATCGCCATCATGGGGTCTACTGGTAAAACCACTAAGGAACTTAAGGATGAGATTTTGGCTCTCAAAGCGAAAGCTGAGGGAGACGGTAAGAAGACTGGCGAGGTACATGCTTTAATgg                                                                                                                                                                                      <  1:100493/301‑1 (MQ=255)`  
                                                                                                                       `ataGAAGAGAACATGGAGGGCATAGTAGGTATCGAGAATAACTCATTCCTTGAGGCACGTAACTTGTTTGATTCGGACCTATCCATCACTATGCCAGACGGACAGCAATTCTCAGTGAATTACCTAAGGGACTTCGATATGTTCCGCATCATGCCAGCGTATGACCGCCGTGTCAATGGTGACATCGCCATCATTGGGTCTACTGGTAAAACCACTAAGGAACTTAATGATGAGATTTTGGCTCTCAAAGCGAAAGCTTAGGGAGACGGTAAGAAGACTGGCGAGGTACATGCTTTAATgg                                                                                                                                                                                      <  1:87815/301‑1 (MQ=255)`  
                                                                                                                           `gagagAACATTGAGGGCTTAGTAGGTATCGAGAATAACTCATTCCTTGAGGCACGTAACTTGTTTGATTCGGACCTATCCATCACTATGCCAGACGGACAGCAATTCTCAGTGAATGACCTAAGGGACTTCGATATGTTCCGCATCATGCCAGCGTATGACCGCCGTGTCAATGGTGGCATCGCCATCATGGGGTCTACTGGTAAAACCACTAAGGAACTTAAGGATGAGATTTTGGCTCTCAAAGCGAAAGCTGAGTGAGACGGTAAGAAGACTGGCGAGGTACATGCTTTAATGGATAc                                                                                                                                                                                  <  1:25531/300‑1 (MQ=255)`  
                                                                                                                           `aagaGAACATTGAGCGCTTAGTAGGTATCGAGAATAACTCATTCCTTGAGGCACGTAACTTGTTTGATTCGGACCTATCCATCACTATGCCAGACGGACAGCAATTCTCAGTGAATGACCTAAGGGACTTCGATATGTTCCGCATCATGCCAGCGTATGACCGCCGTGTCAATGGTGACATCGCCATCATGGGGTCTACTGGTAAAACCACTAAGGAACTTAAGGATGAGATTTTGGCTCTCAAAGCGAAAGCTGAGGGAGACGGTAAGAAGACTGGCGAGGTACATGCTTTAATGGATAc                                                                                                                                                                                  <  1:32466/301‑1 (MQ=255)`  
                                                                                                                              `agaACATTGAGGGCTTAGTAGGTATCGAGAATAACTCATTCCTTGAGGCACGTAACTTGTTTGATTCGGACCTATCCATCACTATGCCAGACGGACAGCAATTCTCAGTGAATGACCTAAGGGACTTCGATATGTTCCGCATCATGCCAGCGTATGACCGCCGTGTCAATGGTGACATCGCCATCATGGGGTCTACTGGTAAAACCACTAAGGAACTTAATGATGAGATTTTGGCTCTCAAAGCGAAAGCTGAGGGAGACGGTAAGAAGACTGGCGAGGTACATGCTTTAATGGATACCGt                                                                                                                                                                               <  1:297857/301‑1 (MQ=255)`  
                                                                                                                              `agaACATTGAGGGCTTAGTAGGTATCGAGAATAACTCATTCCTTGAGGCACGTAACTTGTTTGATTCGGACCTATCCATCACTATGCCAGACGGACAGCAATTCTCAGTGAATGACCTAAGGGACTTCGATATGTTCCGCATCATGCCAGCGTATGACCGCCGTGTCAAGGGTGGCATCGCCATCATGGTGTCTACTGGTAAAACCACTAAGGAACTTAAGGATGAGATTTTGGCTCTCAAAGCGAAAGCTGAGGGAGAGGGTAAGAA‑AATGGCGAGGttccatcttttattggtaacggt                                                                                                                                                                              >  1:23598/1‑279 (MQ=255)`  
                                                                                                                                  `aaTTGAGGGCTTAGTAGGTATCGAGAATAACTCATTCCTTGAGGCACGTAACTTGGTTGATTCGGACCTATCCATCACTATGCCAGACGGACAGCAATTCTCAGTGAATGACCTAATGGACTTCGATAGGTTCCGCATCATGCCAGCGGATGACCGCCGTGTCAATGGTGACATCGCCATCATGGGGTCTACTGGTAAAACCACTAAGGAACTTAAGGATGAGATTTTGGCTCTCAAAGCGAAAGCTGAGGGAGACGGTAAGAAGACTGGCGAGGTACATGCTTTAATGGATACCGTTAAg                                                                                                                                                                           <  1:140090/300‑1 (MQ=255)`  
                                                                                                                                   `atttatggCTTAGTAGGGATCGAGAATAACTCATATCTTGAGGCACGTAACTTGTTGGATTCGGACCTATCCATCACTATGCCAGACGGACAGCAATTCTCAGTGAATGACCTAAGGGACTTCGATATGTTCCGCATCATGCCAGCGTATGACCGCCGTGTCAAGGGTGACATCGCCATCATGGGGACTACTGGTAAAACCTCTAAGGAACTTAAGGATGAGATTTTGGCTCTCAAAGCGAAAGCTGAGGGAGACGGTAAGAAGACTGGCGAGGTACATGCTTTAATGGATACCGTTAAGa                                                                                                                                                                          <  1:146451/295‑1 (MQ=255)`  
                                                                                                                                   `aTTGAGGGCTTAGTAGGTATCGAGAATAACTCATTCCTTGAGGCACGTAACTTGTTTGATTCGGACCTATCCATCACTATGCGAGACGGACAGCAATTCTCAGTGAATGACCTAAGGGACTTCGATATGTTCCGCATCATGCCAGCGTATGACCGCCGTGTCAATGGTGACATCGCCATCATGGGGTCTACTGGTAAAACCACTAAGGAACTTAAGGATGAGATTTTGGCTCTCAAAGCGAAAGCTGAGGGAGACGGTAAGAAGACTGGCGAGGTACATGCTTTAATGGATACCGTTAAGa                                                                                                                                                                          <  1:82609/301‑1 (MQ=255)`  
                                                                                                                                   `aTTGAGGGCTTAGTAGGTATCGAGAATAACTCATTCCTTGAGGCACGTAACTTGTTTGATTCGGACCTATCCATCACTATGCCAGACGGACAGCAATTCTCAGTGAATGACCTAAGGGACTTCGATATGTTCCGCATCATGCCAGCGTATTACCGCCGTGTCAATGGTGGCATCGCCATCATGGGGTCTACTGGTAAAACCACTAAGGAACTTAAGGATGAGATTTTGGCTCTCAAAGCGAAAGCTGAGGGAGACGGTAAGAAGACTGGCAAGGTACATGCTTTAATGGATACCGTTAAGa                                                                                                                                                                          <  1:188795/301‑1 (MQ=255)`  
                                                                                                                                          `gcgtagtagGTATCGAGAATAACTCATTCCTTGAGGCACGTAACTTGTTTGATTCGGACCTATCCATCACTATGCCAGACGGACAGCAATTCTCAGTGAATGACCTAAGGGACTTCGATATGTTCCGCATCATGCCAGCGTATGACCGCCGTGTCAATGGTGACATCGCCATCATGGGGTCTACTGGTAAAACCACTAAGGAACTTAAGGATGAGATTTTGGCTCTCAAAGCGAAAGCTGAGGGAGACGGTAAGAAGACTGGCGAGGTACATGCTTTAATGGATACCGTTAAGATTCTTAc                                                                                                                                                                   >  1:4161/4‑301 (MQ=255)`  
                                                                                                                                          `gCTTAGTAGGTATCGAGAATAACTCATTCCTTGAGGCACGTAACTTGTTTGATTCGGACCTATCCATCACTATGCCAGACGGACAGCAATTCTCAGTGAATGACCTAAGGGACTTCGATATGTTCCGCATCATGCCAGCGTATGACCGCCGTGTCAATGGTGACATCGCCATCATTGGGTCTACTGGTAAAACCACTAAGGAACTTAAGGATGAGATTTTGGCTCTCAAAGCGAAAGCTGAGGGAGACGGTAAGAAGACTGGCTAGGTACATGCTTTAATgctgtctcttatacacatctc                                                                                                                                                                   >  1:20518/1‑281 (MQ=255)`  
                                                                                                                                          `gCTTAGTAGGTATCGAAAATAACTCATTCCTTAAGGCACGTAACTTGTTTGATTCGGACCTATCCATCACTATGCCAGACGGACAGCAATTCTCAGTGAATGACCTAAGGGACTTCGATATGTTCCGCATCATGCCAGCGTATGACCGCCGTGTCAATGGTGGCATCGCCATCATGGGGTCTACTGGTAAAACCACTAAGGAACTTAAGGATGAGATTTTGGCTCTCAAAGCGAAAGCTGAGGGAGACGGTAAGAAGACTGGCGAGGTACATGCTTTAATGGATACCGTTAAGATTCTTAc                                                                                                                                                                   <  1:72413/301‑1 (MQ=255)`  
                                                                                                                                           `cTTAGTAGGTATCGAGAATAACTCATTCCTTGAGGCACGTAACTTGTTTGATTCGGACCTATCCATCACTATGCCAGACGGACAGCAATTCTCAGTGAATGACCTAAGGGACTTCGATATGTTCCGCATCATGCCAGCGTATGACCGCAGTGTCAATGGTGACATCGCCATCATGGGGTCTACTGGTAAAACCACTAAGGAACTTAAGGATGAGATTTTGGCTCTCAAAGCGAAAGCTGAGGGAGACGGTAAGAAGACTGGCGAGGTACATTCTTTAATGGATACCGTTAAGATTCTTaat                                                                                                                                                                  >  1:181875/1‑299 (MQ=255)`  
                                                                                                                                                     `aTCGAGAATAACTCATTCCTTGAGGCACGTAACTTGTTTGATTCGGACCTATCCATCACTATGCCAGACGGACAGCAATTCTCAGTGAATGACCTAAGGGACTTCGATATGTTCCGCATCATGCCAGCGTATGACCGCCGTGTCAATTGTGACATCGCCATCATGGGGTCTACTGGTAAAACCACTAAGGAACTTAAGGATGAGATTTTGGCTCTCAAAGCGAAAGCTGAGGGAGACGGTAAGAAGACTGGCGAGGTACATGCTTTAATGGATACCGTTAAGATTCTTACTGGTCGTGCTa                                                                                                                                                        >  1:85266/1‑301 (MQ=255)`  
                                                                                                                                                        `gagaATAACTCATTCCTTGAGGCACGTAACTTGTTTGATTCGGACCTATCCATCACTATGCCAGACGGACAGCAATTCTCAGTGAATTACCTAAGGGACTTCGATATGTTCCGCATCATGCCAGCGTATGACCGCCGTGTCAATGGTGACATCGCCATCATGGGGTCTACTGGTAAAACCACTAAGGAACTTAATGATGAGATTTTGGCTCTCAAAGCGAAAGCTGAGGGAGACGGTAAGAAGACTGGCGAGGTACATGCTTTAATGGATACCGTTAAGATTCTTACTGGTCGTGCTAGAc                                                                                                                                                     >  1:300780/1‑301 (MQ=255)`  
                                                                                                                                                        `gagaATAACTAATTCCTTGAGGCACGTAACTGGTGTGAGTCGGACCTATCCATCACTATGCCAGACGGACAGCAATTCTCAGTGAATGACCTAAGGGACTTCGATATGTTCCGCATCATGCCAGCGTATGACCGCCGTGTCAATGGTTACATCGCCATCATGGGGTCTACTGGTAAAACCACTAAGGAACTTAAGGATGAGATTTTGGCTCTCAAAGCGAAAGCTGAGGGAGACGGTAAGAAGACTGGCGAGGTACATGCTTTAATGGATACCGTTAAGATTCTTACTGGTCGTGCTAGAc                                                                                                                                                     <  1:69634/301‑1 (MQ=255)`  
                                                                                                                                                         `agaATAACTCATTCCTTGAGGCACGTAACTTGTTTGATTCGGACCTATCCATCACTATGCCAGACGGACATCAATTCTCAGTGAATGACCTAAGGGACTTCGATATGTTCCGCATCATGCCAGCGTATGACCGCCATGTCAATGGTTACATCGCCATCATGGGGTCTACTGGTAAAACCACTAAGGAACTTAAGGATGAGATTTTGGCTCTCAAAGCGGAAGCTGAGGGAGACGGTAAGAAGACTGGCGAGGTACATGCTTTAATGGATACCGTTAAGATTCTTACTGGTCGGGCTAGAcc                                                                                                                                                    >  1:234025/1‑300 (MQ=255)`  
                                                                                                                                                              `cgcTCATTCCTTGAGGCACGTAACTTGTTTGATTCGGACCTATCCATCACTATGCCAGACGGACAGCAATTCTCAGTGAATGACCTAAGGGACTTCGATATGTTCCGCATCATGCCAGCGTATGACCGCCGTGTCAATGGTGGCATCGCCATCATGGGGTCTACTGGTAAAACCACTAAGGAACTTAAGGATGAGATTTTGGCTCTCAAAGCGAAAGCTGAGGGAGACGGTAAGAAGACTGGCGAGGTACATGCTTTAATGGATACCGTTAAGATTCTTGCTGGTCGTGCTAGACGCAATc                                                                                                                                               <  1:226648/299‑1 (MQ=255)`  
                                                                                                                                                                `cTCATTCCTTGAGGCACGTAACTTGTTTGATTCGGACCTATCCATCACTATGCCAGACGGACAGCAATTCTCAGTGAATGACCTAAGGGACTTCGATATGTTCCGCATCATGCCAGCGTATGACCGCCGTGTCAATGGTGGCATCGCCATCATGGGGTCTACTGGTAAAACCACTAAGGAACTTAAGGATGAGATTTTGGCTCTCAAAGCGAAAGCTGAGGGAGACGGTAAGAAGACTGGCGAGGTACATGCTTTAATGGATACCGTTAAGATTCTTACTGGTctgtctcttatacacatc                                                                                                                                             >  1:170050/1‑284 (MQ=255)`  
                                                                                                                                                                   `aTTCCTTGAGGCACGTAACTTGTTTGATTCGGACCTATCCATCACTATGCCAGACGGACAGCAATTCTCAGTGAATGACCTAAGGGACTTCGATATGTTCCGCATCATGCCAGCGTATGACCGCCGTGTCAATGGTGACATCGCCATCATGGGGTCTACTGGTAAAACCACTAAGGAACTTAATGATGAGATTTTGGCTCTCAAAGCGAAAGCTGAGGGAGACGGTAAGAAGACTGGCGAGGTACATGCTTTAATGGATACCGTTAAGATTCTTACTGGTCGTGCTAGACGCAATCAGGac                                                                                                                                          <  1:116841/301‑1 (MQ=255)`  
                                                                                                                                                                             `gCACGTAACTTGTTTGATTCGGACCTATCCATCACTATGCCAGACGGACAGCAATTCTCAGTGAATGACCTAAGGGACTTCGATATGTTCCGCATCATGCCAGCGTATGACCGCCGTGTCAATGGTGGCATCGCCATCATGGGGTCTACTGGTAAAACCACTAAGGAACTTAAGGATGAGATTTTGGCTCTCAAAGTGAAAGCTGAGGGAGACGGTAAGAAGACTGGCGAGGTACATGCTTTAATGGATACCGTTAAGATTCTTACTGGTCGTGCTAGACGCAATCAGGACACTGTGTggg                                                                                                                                <  1:103096/301‑1 (MQ=255)`  
                                                                                                                                                                             `gCACGTAACTTGTTGGATTCGGACCTATCCATCACTATGCCAGACGGACAGCAATTCTCAGTGAATGACCTAAGGGACTTCGATATGTTCCGCATCATGCCAGCGTATGACCGCCGTGTCAATGGTGGCATCGCCATCATGGGGTCTACTGGTAAAACCACTAAGGAACTTAAGGATGAGATTTTGGCTCTCAAAGCGAAAGCTGAGGGAGACGGTAAGAAGACTGGCGAGGTACATGCTTTAATGGATACCGTTAAGATTCTTACTGGTCGTGCTAGACGCAATCAGGACACTGTGTggg                                                                                                                                <  1:174020/301‑1 (MQ=255)`  
                                                                                                                                                                               `tgtataagagacagATTCGGACCTATCCATCACTATGCCAGACGGACAGCAATTCTCAGTGAATGACCTAAGGGACTTCGATATGTTCCGCATCATGCCAGCGTATGACCGCCGTGTCAATGGTGACATCGCCATCATGGGGTCTACTGGTAAAACCACTAAGGAACTTAATGATGAGATTTTGGCTCTCAAAGCGAAAGCTGAGGGAGACGGTAAGAAGACTGGCGAGGTACATGCTTTAATGGATACCGTTAAGATTCTTACTGGTCGTGCTAGACGCAATCAGGACACTGTGTGGGaa                                                                                                                              <  1:271807/288‑1 (MQ=255)`  
                                                                                                                                                                               `ggtataagagacagtttCGGACCTATCCATTACTATGCCAGCCGGACAGTAATTCTCAGTGAATGACCTCAGGGACTTCGATATGTTCCGCATCATGCCAGCGTATGACCGCCGTGTCAATTGTGACATCGCCATCATGGGGTCTACTGGTAAAACCACTAATGAACTTAAGGATGAGATTTTGGCTCTCAAAGCGACAGCTGAGGGAGACGGTAAGAAGACTGGCGAGGTACATGCTTTAATGGATACCGTTAAGATTCTTACTGGTCGTGCTAGACGCAATCAGGACACTGTGTGGGaa                                                                                                                              <  1:134010/286‑1 (MQ=255)`  
                                                                                                                                                                               `aCGTAACTTGTTTGATTCGGACCTATCCATCACTATGCCAGACGGACAGCAATTCTCAGTGAATGACCTAAGGGACTTCGATATGTTCCGCATCATGCCAGCGTATGACCGCCGTGTCAATTGTGACATCGCCATCATGGGGTCTACTGGTAAAACCACTAAGGAACTTAAGGATGAGATTTTGGCTCTCAAAGCGAAAGCTGAGGGAGACGGTAAGAAGACTGGCGAGGTACATGCTTTAATGGATACCGTTAAGATTCTTACTGGTCGTGCTAGACGCAATCAGGACACTGTGTGGGaa                                                                                                                              <  1:51337/301‑1 (MQ=255)`  
                                                                                                                                                                               `aCGTAACTGGTTTGAGTTGGACCGAGCCAGTACTAGGCCAGACGGACAGCAATTCTCAGTGAATGACCTAAGGGACTTAGATATGTTACGCATCATGCCAGCGTAGGACCGCCGTGTCAAGGGTGACATCGACATCATGGGGTCTACGGGGAAAACCACTAAGGAACTTAATGATGAGATGTTGGCTCTCAAAGCGAAAGCTGAGGGAGACGGTAAGAAGAATGGCGAGGGACATGCTGTAAGGGATAACGTTAAGATTCGTACTGGTCGTGCTAGACGCAATCAGGACACTGTGTGGGaa                                                                                                                              <  1:36485/301‑1 (MQ=255)`  
                                                                                                                                                                                `cGTAACTTGTTTGAATCGGACCTACCCATCACTATGCCAGACGGACCGCAATTCTCAGTGAATGACCTAAGGTACTTCGATATGTTCCGCATCATGCCAGCGTATTACCGCCGTGTCAATGGTGGCATCGCCATCATGGGGTATACTGGTAAAACCACTAAGGAACTTAAGGATGAGATTTTGGCTCTCAAAGCGAAAGCTGAGGGAGACGGTAAGAAGACTGGCGAGGTACATGCTTTAATGGATACCGTTAAGATTCTTACTGGTCGTGcagtaagaatcttaacggtatccattaaag                                                                                                                             <  1:90733/301‑30 (MQ=255)`  
                                                                                                                                                                                       `tGTTTGATTCGGACCTATCCATCACTATGCCAGACGGACAGCAATTCTCAGTGAATGACCTAAGGGACTTCGATATGTTCCGCAACAGGCCAGCGTATGACCGCCGTGTCAATGGTGACATCGCCATCAT‑GGGTCTACTGGTAAAACCACTAAGGAACTTAAGGATGAGATTTTGGCTCTCAAAGCGAAAGCTGAGGGAGACGGTAAGAAGACTGGCGAGGTACATGCTTTAATGGATACCGTTAAGATTCTTACTGGTCGTGCTAGACGCAATCAGGACACTGTGTGGGAAACCTCACTg                                                                                                                     <  1:105349/301‑1 (MQ=255)`  
                                                                                                                                                                                          `gagacagggACCTATCCATCACTATGCCAGACGGACAGCAATTCTCAGTGAATGACCTAAGGGACTTCGATATGTTCCGCATCATGCCAGCGTATGACCGCCGTGTCAATGGTGACATCGCCATCATGGGGTCTACTGGTAAAACCACTAAGGAACTTAATGATGAGATTTTGGCTCTCAAAGCGAAAGCTGAGGGAGACGGTAAGAAGACTGGCGAGGTACATGCTTTAATGGATACCGTTAAGATTCTTACTGGTCGTGCTAGACGCAATCAGGACACTGTGTGGGAAACCTCACTGCg                                                                                                                   <  1:131565/294‑1 (MQ=255)`  
                                                                                                                                                                                                    `ccTATCCATCACTATGCCAGACGGACAGCAATTCTCAGTGAATGACCTAAGGGACTTCGATATGTTCTGCATCATGCCAGCGTATTACCGCCGTGTCAATGGTGGCATCGCCATCATGGGGTCTACTGGTAAAACCACTAAGGAACTTAAGGATGAGATTTTGGCTCTCAAAGCGAAAGCTTAGTGAGACGGTAAGAAGACTGTCGAGGTACATGCTTTAATGGATACCGTTAAGATTCTTACTGGTCGTGCTAGACGCAATCAGGCCACtgtgctgtctcttatacacatctccgagccc                                                                                                         >  1:110815/1‑274 (MQ=255)`  
                                                                                                                                                                                                       `acccATCACTATGCCAGACGGACAGCAATTCTCAGTGAATGACCTAAGGGACTTCGATATGTTCCGCATCATGCCAGCGTATGACCGCCGTGTCAATGGTGACATCGCCATCATGGGGTCTACTGGTAAAACCACTAAGGAACTTAAGGATGAGATTTTGGCTCTCAAAGCGAAAGCTGAGGGAGACGGTAAGAAGACTGGCGAGGTACATGCTTTAATGGATACAGTTAAGATTCTTACTGGTCGTGCTAGACGCAATCAGGACACTGTGTGGGAAACCTCACTGCGTGCCATCAAGGAc                                                                                                      <  1:125301/299‑1 (MQ=255)`  
                                                                                                                                                                                                       `aTCCATCACTATGCCAGACGGACAGCAATTCTCAGTGAATGACCTAAGGGACTTCGATATGTTCCGCATCATGCCAGCGTATGACCGCCGTGTCAATGGTGGCATCGCCATCATGGGGTCTACTGGTAAAACCACTAAGGAACTTAAGGATGAGATTTTGGCTCTCAAAGCGAAAGCTGAGGGAGACGGTAAGAAGACTGGCGAGGTACATGCTTTAATGGATACCGTTAAGATTCTTACTGGTCGTGCTAGACGCAATCAGGACACTGTGTGGGTAACCTCACTGCGTGCCATCAATGAc                                                                                                      >  1:223822/1‑301 (MQ=255)`  
                                                                                                                                                                                                       `aTCCATCACTATGCCAGACGGACAGCAATTCTCAGTGAATGACCTAAGGGACTTCGATATGTTCCGCATCATGCCAGCGTATGACCGCCGTGTCAATGGTGGCATCGCCATCATGGGGTCTACTGGTAAAACCACTAAGGAACTTAAGGATGAGATTTTGGCTCTCAAAGCGAAAGCTGAGGGAGACGGTAAGAAGACTGGCGAGGTACATGCTTTAATGGATACCGTTAAGATTCTTACTGGTCGTGCTAGACGCAATCAGGACACTGTGTGGGAAACCTCACTGCGTGCCATCAATGAc                                                                                                      >  1:11496/1‑301 (MQ=255)`  
                                                                                                                                                                                                       `aTCCATCACTATGCCAGACGGACAGCAATTCTCAGTGAATGACCTAAGGGACTTCGATATGTTCCGCATCATGCCAGCGTATGACCGCCGTGTCAATGGTGACATCGCCATCATGGGGTCTACTGGTAAAACCACTAAGGAACTTAAGGATGAGATTTTGGCTCTCAAAGCGAAAGCTGAGGGAGACGGTAAGAAGACTGGCGAGGTACATGCTTTAATGGATACCGTTAAGATTCTTACTGGTCGTGCTAGACGCAATCAGGACACTGTGTGGGaactgtctcttatacacatctccgag                                                                                                      >  1:262412/1‑277 (MQ=255)`  
                                                                                                                                                                                                        `tCCATCACTATGCCAGACGGACAGGAATTCTCAGTGAATGACCTAAGGGACTTCGATATGTTCCGCATCATGCCAGCGTATGACCGCCGTGTCAATGGTGGCATCGCCATCATGGGGTCTACTGGTAAAACCACTAAGGAACTTAAGGATGAGATTTTGGCTCTCAAAGCGAAAGCTGAGGGAGACGGTAAGAAGACTGGCGAGGTACATGCTTTAATGGATACCGTTAAGATTCTTACTGGTCGTGCTAGACGCAATCAGGACACTGTGTGGGAAACCTCACTGCGTGCCATCAATGAcc                                                                                                     <  1:191547/301‑1 (MQ=255)`  
                                                                                                                                                                                                        `aagagacagaTGCCAGACGGACAGCAATTCTCAGTGAATGACCTAAGGGACTTCGATATGTTCCGCATCATGCCAGCGTACGACCGCCGTGTCAATGGTGGCATCGCCATCATGGGGTCTACTGGTAAAACCACTAAGGAACTTAAGGATGAGATTTTGGCTCTCAAAGCGAAAGCTGAGGGAGACGGTAAGAAGACTGGCGAGGTACATGCTTTAATGGATACCGTTAAGATTCTTACTGGTCGTGCTAGACGCAATCAGGACACTGTGTGGGAAACCTCACTGCGTGCCATCAATGAcc                                                                                                     <  1:315400/292‑1 (MQ=255)`  
                                                                                                                                                                                                            `tCACTATGCCAGACGGACAGCAATTCTCAGTGAATGACCTAAGGGACTTCGATATGTTCCGCATCATGCCAGCGTATGACCGCCGTGTCAATGGTGACATCGCCATCATGGGGTCTACTGGTAAAACCACTAAGGAACTTAATGATGAGATTTTGGCTCTCAAAGCGAAAGCTGAGGGAGACGGTAAGAAGACTGGCGAGGTACATGCTTTAATGGATACCGTTAAGATTCTTACTGGTCGTGCTAGACGCAATCAGGCCACTGTGTGGGAAACCTCACTGCGTGCCATAAATGACCTAgg                                                                                                 >  1:129221/1‑301 (MQ=255)`  
                                                                                                                                                                                                            `tCACTATGCCAGACGGACAGCAATTCTAAGTGAATGACCTAAGGGACTTCGATATGTTCCGCATCATGCCAGCGTATGACCGCCGTGTCAATGGTGACATCGCCATCATGGGGTCTACTGGTAAAACCACTAAGGAACTTAAGGATGAGATTTTGGCTCTCAAAGCGAAAGCTGAGGGAGACGGTAAGAAGACTGGCGAGGTACATGCTTTAATGGATACCGTTAAGATTCTTACTGGTCGTGCTAGACGCAATCAGGACACTGTGTGGGAAACCTCACTGCGTGCCATCAATGACCTAgg                                                                                                 >  1:20287/1‑301 (MQ=255)`  
                                                                                                                                                                                                             `cACTATGCCAGACGGACAGCAATTCTCAGTGAATGACCTAAGGGACTTCGATATGTTCCGCATCATGCCAGCGTATGACCGCCGTGTCAATGGTGACATCGCCATCATGGGGTCTACTGGTAAAACCACTAAGGAACTTAAGGATGAGATTTTGGCTCTCAAAGCGAAAGCTGAGGGAGACGGTAAGAAGACTGGCGAGGTACATGCTTTAATGGATACCGTTAAGATTCTTACTGGTCGTGCTAGACGCAATCAGGACACTGTGTGGGAAACCTCACTGCGTGCCATCAATGACCTAggg                                                                                                >  1:249035/1‑301 (MQ=255)`  
                                                                                                                                                                                                                `tcggagatgtgtataagagacagcAGGGAATGACCTAAGGGACTTCGATATGTTCCGCATCATGCCAGCGTATGACCGCCGTGTCAATGGTTACATCGCCATCATGGGGTCTACTGGTAAAACCACTAAGGAACTTAAGGATGAGATTTTGGCTCTCAAAGCGAAAGCTGAGGGAGACGGTAAGAAGACTGGCGAGGTACATGCTTTAATGGATACCGTTAAGATTCTTACTGGTCGTGCTAGACGCAATCAGGACACTGTGTGGGAAACCTCACTGCGTGCCATCAATGACCTAGGGttc                                                                                             <  1:295522/278‑1 (MQ=255)`  
                                                                                                                                                                                                                 `aTGCCAGACGGACAGCAATTCTCAGTGAATGACCTAAGGGACTTCGATATGTTCCGCATCATGCCAGCGTATGACCGCCGTGTCAATGGTGACATCGCCATCATGGGGTCTACTTGTAAAACCACTAAGGAACTTAAGGATGAGATTTTGGCTCTCAAAGCGAAAGCTGAGGGAGACGGTAAGAAGACTGGCTAGGTACATGCTTTAATGGATACCGTTAAGATTCTTACTGGTCGTGCTAGACGCAATCAGGACACTGTGTGGGAAACCTCACTGCGTGCCATCAATGACCTAGGGttct                                                                                            >  1:107581/1‑301 (MQ=255)`  
                                                                                                                                                                                                                 `aTGCCAGACGGACAGCAATTCTCAGTGAATGACCTAAGGGACTTCGATATGTTCCGCATCATGCCAGCGTATGACCGCCGTGTCAATGGTGACATCGCCATCATGGGGTCTACTGGTAAAACCACTAAGGAACTTAATGATGAGATTTTGGCTCTCAAAGCGAAAGCTGAGGGAGACGGTAAGAAGACTGGCGAGGTACATGCTTTAATGGATACCGTTAAGATTCTTAATGGTCGTGCTAGACGCAATCAGGACACTGTGTGGGAAACCTCACTGCGTGACATCAATGACCTAgggctgt                                                                                            >  1:64073/1‑297 (MQ=255)`  
                                                                                                                                                                                                                                       `tcggagatgtgtataagagacaggATATGTTCCGCATCATGCCAGCGTATGACCGCCGTGTCAATGGTGACATCGCCATCATGGGGTCTACTGGTAAAACCACTAAGGAACTTAATGATGAGATTTTGGCTCTCAAAGCGAAAGCTGAGGGAGACGGTAAGAAGACTGGCGAGGTACATGCTTTAATGGATACCGTTAAGATTCTTACTGGTCGTGCTAGACGCAATCAGGACACTGTGTGGGAAACCTCACTGCGTGCAATCAATGACCTAGGGTTCTTCGCTAAGAACGCCTACATggg                                                                      <  1:93870/278‑1 (MQ=255)`  
                                                                                                                                                                                                                                       `cAGTGAATGACCTAAGGGACTTCGATATGTTCCGCATCATGCCAGCGTATGACCGCCGTGTCAATGGTGACATCGCCATCATGGGGTCTACTGGTAAAACCACTAAGGAACTTAAGGATGAGATTTTGGCTCTCAAAGCGAAAGCTGAGGGAGACGGTAAGAAGACTGGCGAGGTACATGCTTTAATGGATACCGTTAAGATTCTTACTGGTCGTGCTAGACGCAATCAGGACACTGTGTGGGAAACCTCACTGCGTGCCATCAATGACCTAGGGTTCTTCGCTAAGAACGCCTACATggg                                                                      >  1:269675/1‑301 (MQ=255)`  
                                                                                                                                                                                                                                           `gAATGACCTAAGGGACTTCGATATGTTCCGCATCATGCCAGCGTATGACCGCCGTGTCAATTGTGACATCGCCATCATGGGGTCTACTGGTAAAACCACTAAGGAACTTAAGGATGAGATTTTGGCTCTCAAAGCGAAAGCTGAGGGAGACGGTAAGAAGACTGGCGAGGTACATGCTTTAATGGATACCGTTAAGATTCTTACTGGTCGTGCTAGACGCAATCAGGACACTGTGTGGGAAACCTCACTGCGTGCCATCAATGACCTAGGGTTCTTCGCTAAGAACGCCTACATGGGTGCt                                                                  >  1:215280/1‑301 (MQ=255)`  
                                                                                                                                                                                                                                              `tGACCCAAGGGCTTTCTATACCTTCCGCATCATGCCAGCGTATTACCGCCGTGTCAATGTTTACAGCGCCATCATGGGGTCTACTTGTAAAACCACTAAGGAACTTAAGGATGAGATTTTGGCTCTCAAAGCGAAAGCTGAGGGAGACGGTAAGAAGACTGGCGAGGTACATGCTTTAATGGATACCGTTAAGATTCTTTCTGGTCGTGCTAGACGCAATCAGGATACTGTGTGGGAAACCTCACTGCGTGCCATCAATGACCTAGGGTTCTTCGCTAAGAACGCCTACATGGGTGCTCAg                                                               <  1:307081/301‑1 (MQ=255)`  
                                                                                                                                                                                                                                               `gACCTAAGGGACTTCGATATGTTCCGCATCATGCCAGCGTATGACCGCCGTGTCAATGGTGACATCGCCATCATGGGGTCTACTGGTAAAACCACTAAGGAACTTAATGATGAGATTTTGGCTCTCAAAGCGAAAGCTGAGGGAGACGGTAAGAAGACTGGCGAGGTACATGCTTTAATGGATACCGTTAAGATTCTTACTGGTCGTGCTAGACGCAATCAGGACACTGTGTGGGAAACCTCACTGCGTGCCATCAATGACCTAGGGTTCTTCGCTAAGAACGCCTACATGGGTGCTCAGa                                                              >  1:57488/1‑301 (MQ=255)`  
                                                                                                                                                                                                                                                 `ccTAAGGGACTTCGATATGGTCCGCATCATGCCAGCGTATGACCGCCGTGTCAATGGTGACATCGCAATCATGGGGTCTACTGGTAAAACCACTAAGGAACTTAATGATGAGATTTTGGCTCTCAAAGCGAAAGCTGAGGGAGACGGTAAGAAGACTGGCGAGGTACATGCTTTAATGGATACCGTTAAGATTCTTACTGGTCGTGCTAGACGCAATCAGGACACTGTGTGGGAAACCTCACTGCGTGCCATCAATGACCTAGGGTTCTTCGCTAAGAACGCCTACATGGGTGCTCAGAAc                                                            <  1:310032/301‑1 (MQ=255)`  
                                                                                                                                                                                                                                                      `gggACTTCGATATGTACCGCATCATGCACGCGTATGACCGCCGTGTCAATGGTGACAGCGCCATCATGGGGTCTACTGGTAAAACCACTAAGGAACTTAAGGATGAGATTTTGGCTCTCAAAGCGAAAGCTGAGGGAGACGGTAAGAAGACTGGCGAGGTACATGCTTTAATGGATACCGTTAAGATTCTTACTGGTCGTGCTAGACGCAATCAGGACACTGTGTGGGAAACCTCACTGCGTGCCATCAATGACCTAGGGTTCTTCGCTAAGAACGCCTACATGGGTGCTCAGAACATTAc                                                       <  1:74576/301‑1 (MQ=255)`  
                                                                                                                                                                                                                                                        `gACTTCTATATGTTCCGCATCATGCCAGCGTATGACCGCCGTGTCAATGGTGACATCGCCATCATGGGGTCTACTGGTAAAACCACTAAGGAACTTAATGATGAGATTTTGGCTCTCAAAGCGAAAGCTGAGGGAGACGGTAAGAAGACTGGCGAGGTACATGCTTTAATTTATACCGTTAAGATTCTTACTGGTCGTGCTAGACGCAATCAGGACACTGTGTGGGAAACCTCACTGCGTGCCATCAATGACCTAGGGTTCTTCGCTAAGAACGCCTACATGGGTGCTCAGAACATTACgg                                                     >  1:328197/1‑301 (MQ=255)`  
                                                                                                                                                                                                                                                        `gACTTCGATATGTTCCGCATCATGCCAGCGTATGACCGCAGTGTCAATGGTGACATCGCCATCATGGGGTCTACTGGTAAAACCACTAAGGAACTTAAGGATGAGATTTTGGCTCTCAAAGCGAAAGCTGAGGGAGACGGTAAGAAGAATGGCGAGGTACATGCTTTAATGGATACCGTTAAGATTCTTACTGGTCGTGCTAGACGCAATCAGGACACTGTGTGGGAAACCTCACTGAGTGCCATCAATGACCTAGGGTTCTTCGCTAAGAACGGCAACATGGGTGCTCAGAACATTACgg                                                     >  1:313695/1‑301 (MQ=255)`  
                                                                                                                                                                                                                                                          `cTTCGATATGTTCCGCATCATGCCAGCGTATGACCGCCGTGTCAATGGTGACATCGCCATCATGGGGTCTACTGGTAAAACCACTAAGGAACTTAAGGATGAGATTTTGGCTCTCAAAGCGAAAGCTGAGGGAGACGGTAAGAAGACTGGCGAGGTACATGCTTTAATGGATACCGTTAAGATTCTTACTGGTCGTGCTAGACGCAATCAGGACACTGTGTGGGAAACCTCACTGCGTGCCATCAATGACCTAGGGTTCTTCGCTAAGAACGCCTACATGGGTGCTCAGAACATTACGgag                                                   >  1:33785/1‑301 (MQ=255)`  
                                                                                                                                                                                                                                                            `tCGATATGTTCCGCATCATGCCAGCGTATGACCGCCGTGTCAATGGTGACATCGCCATCATGGGGTCTACTGGTAAAACCACTAAGGAACTTAAGGATGAGATTTTGGCTCTCAAAGCGAAAGCTGAGGTAGACGGTAAGAAGACTGGCGAGGTACATGCTTTAATGGATACCGTTAAGATTCTTACTGGTCGTGCTAGACGCAATCAGGACACTGTGTGGGAAACCTCACTGCGTGCCATCAATGACCTAGGGTTCTTCGCTAAGAACGCCTACATGGTTGCTCAGAACATTACGGAGAt                                                 >  1:8031/1‑301 (MQ=255)`  
                                                                                                                                                                                                                                                             `cGATATGTTCCGCATCATGCCAGCGTATGACCGCCGTGTCAATGGTGACATCGCCATCATGTGGTCTACTGGTAAAACCACTAAGGAACTTAAGGATGAGATTTTGGCTCTCAAAGCGAAAGCTGAGGGAGACGGTAAGAAGACTGGCGAGGTACATGCTTTAATGGATACCGTTAAGATTCTTACTGGTCGTGCTAGACGCAATCAGGACACTGTGTGGGAAACCTCACTGCGTGCCATCAATGACCTAGGGTTCTTCGCTAAGAACGCCTACATGGGTGcctgtctcttatacacatct                                                >  1:274206/1‑282 (MQ=255)`  
                                                                                                                                                                                                                                                                 `atGTTCCGCATCATGCCAGCGTATGACCACCGTGTCAATGGTGACATCGCCATCATGGGGTCTACTGGTAAAACCACTAAGGAACTTAAGGATGAGATTTTGGCTCTCAAAGCGAAAGCTGAGGGAGACGGTAAGAAGACTGGCGAGGTACATGCTTTAATGGATACCGTTAAGATTCTTACTGGTCGTGCTAGACGCAATCAGGACACTGTGTGGGAAACCTCACTGCGTGCCATCAATGACCTAGGGTTCTTCGCTAAGAACGCCTACATGGGTGCTCAGAACATTACGGAGATTGCTg                                            >  1:276987/1‑301 (MQ=255)`  
                                                                                                                                                                                                                                                                    `ttCCGCATCATGCCAGCGTATGACCGCCGTGTCAATGGTGACATCGCCATCATGGGGTCTACTGGTAAAACCACTAAGGAACTTGAGGATGAGATTTTGGCTCTCAAAGCGAAAGCTGAGGGAGACGGTAAGAAGACTGGCGAGGTACATGCTTTAATGGATACCGTTAAGATTCTTACTGGTCGTGCTAGACGCAATCAGGACACTGTGTGGGAAACCTCACTGCGTGCCATCAATGACCTAGGGTTCTTCGCTAAGAACGCCTACATGGGTGCTCAGAACATTACGGAGATTGCTGGga                                         <  1:291672/301‑1 (MQ=255)`  
                                                                                                                                                                                                                                                                         `catcatGCCAGCGTATGACCGCCGTGTCAATGGTGGCATCGCCATCATGGGGTCTACTGGTAAAACCACTAAGGAACTTAAGGATGAGATTTTGGCTCTCAAAGCGAAAGCTGAGGGAGACGGTAAGAAGACTGGCGAGGTACATGCTTTACTGGATACCGTTAAGATTCTTACTGGTCGTGCTAGACGCAATCATGACACTGTGTGGGAAACCTCACTGCGTGCCATCAATGACCTAGGGTTCTTCGCTCAGAACGCCTACATGGGTGCTCAGAACATTACGGAGCTTGCTTGGATGAtt                                    >  1:155508/1‑301 (MQ=255)`  
                                                                                                                                                                                                                                                                                   `gCGTATGACCGCAGTGTCAATGGTGACATCGCCATCATGGGGTCTACTGGTAAAACCACTAAGGAACTTAAGGATGAGATTTTGGCTCTCAAAGCGAAAGCTGAGGGAGACGGTAAGAAGACTGGCGAGGTACATGCTTTAATGGATACCGTTAAGATTCTTACTGGTCGTGCTAGACGCAATCAGGACACTGTGTGTGAAACCTCACTGCGTGCCATCAATGACCTAGGGTTCTTCGCTAAGAACGCCTACATGGGTGCTCAGAACATTACGGAGATTGCTGGGATGATTGTCACTGGTa                          >  1:166982/1‑301 (MQ=255)`  
                                                                                                                                                                                                                                                                                        `tGACCGCCGTGTCAATGGTGGCATCGCCATCATGGGGTCTACTGGTAAAACCACTAAGGAACTTAAGGATGAGATTTTGGCTCTCAAAGCGAAAGCTGAGGGAGACGGTAAGAAGACTGGCGAGGTACATGCTTTAATGGATACCGTTAAGATTCTTACTGGTCGTGCTAGACGCAATCAGGACACTGTGTGGGAAACCTCACTGCGTGCCATCAATGACCTAGGGTTCTTCGCTAAGAACGCCTACATGGGTGCTCAGAACATTACGGAGATTGCTGGGATGATTGTCACTGGTAACGtt                     >  1:120994/1‑301 (MQ=255)`  
                                                                                                                                                                                                                                                                                                  `gtCAATGGTGGCATCGCCATCATGGGGTCTACTGGTAAAACCACTAAGGAACTTAAGGATGAGATTTTGGCTCTCAAAGCGAAAGCTGAGGGAGACGGTAAGAAGACTGGCGAGGTACATGCTTTAATGGATACCGTTAAGATTCTTACTGGTCGTGCTAGACGCAATCAGGACACTGTGTGGGAAACCTCACTGCGTGCCATCAATGACCTAGGGTTCTTCGCTAAGAACGCCTACATGGGTGCTCAGAACATTACGGAGATTGCTGGGATGATTGTCACTGGTAACGTTCGTGCTCTAg           <  1:198261/301‑1 (MQ=255)`  
                                                                                                                                                                                                                                                                                                    `aaaTGGTGACATCGCCATCATGGGGTCTACTGGTAAAACCACTAAGGAACTTAAGGATGAGATTTTGGCTCTCAAAGCGAAAGCTGAGGGAGACTGTAAGAAGACTGGCGAGGTACATGCTTTAATGGATACCGTTAAGATTCTTACTGGTCGTGCTAGACGCAATCAGGACACTGTGTGGGAAACCTCACTGCGTGCTATCAATGACCTAGGGTTCTTCGCTAAGAACGCCTACATGGGTGCTCAGAACATTACGGAGATTGCTGGGATGATTGTCACTGGTAACGTTCGTGCTCTAggg         <  1:16319/300‑1 (MQ=255)`  
                                                                                                                                                                                                                                                                                                     `aaTGGTGACATCGCCATCATGGGGTCTACGGGTAAAACCACGAAGGAACTTAATGATGAGATTTTGGCTCTCAAAGCGAAAGCTGAGGGAGACGGTAAGAAGACTGGCGAGGTACATGCTTTAATGGATACCGTTAAGATTCTTACTGGTCGTGCTAGACGCAATCAGGACACTGTGTGGGAAACCTCACTGCGTGCCATCAATGACCTAGGGTTCTTCGCTAAGAACGCCTACATGGGTGCTCAGAACATTACGGAGATTGCTGGGATGATTGTCACTGGTAACGTTCGTGCTCTAGGGc        <  1:62539/301‑1 (MQ=255)`  
                                                                                                                                                                                                                                                                                                          `agACATCGCCATCAAGGGGTCTACTGGTAAAACCACTAAGGAACTTAAGGATGAGATTTTGGCTCTCAAAGCGAAAGCTGAGGGAGACGGTAAGAAAACTGGCGAGGTACATGCTTTAATGGATACCGTTAAGATTCTTACTGGTCGTGCTAGACGCAATCAGGACACTGTGTGGGAAACCTCACTGCGTGCCATCAATGACCTAGGGTTCTTCGCTAAGGACGCCTACATGGGTGCTCAGAACATTACGGAGATTGCTGGGATGATTGTCACTGGTAACGTTCGTGCTCTAGGGCATGGt   <  1:76863/300‑1 (MQ=255)`  

                                                                                                                                                                                                                                                                                                            `|`                                                                                                                                                                                                                                                                                                             
`ATGAGATGCTTAAGGAATTACACGGGGTGAAGGAAGTAACACCAGAGATGGTAGAGAAGTACGCTATGGATAAGGCTTATGGTATCTCCCACTCAGACCAGTTCACCAACAGTTCCATAATAGAAGAGAACATTGAGGGCTTAGTAGGTATCGAGAATAACTCATTCCTTGAGGCACGTAACTTGTTTGATTCGGACCTATCCATCACTATGCCAGACGGACAGCAATTCTCAGTGAATGACCTAAGGGACTTCGATATGTTCCGCATCATGCCAGCGTATGACCGCCGTGTCAATGGTGACATCGCCATCATGGGGTCTACTGGTAAAACCACTAAGGAACTTAAGGATGAGATTTTGGCTCTCAAAGCGAAAGCTGAGGGAGACGGTAAGAAGACTGGCGAGGTACATGCTTTAATGGATACCGTTAAGATTCTTACTGGTCGTGCTAGACGCAATCAGGACACTGTGTGGGAAACCTCACTGCGTGCCATCAATGACCTAGGGTTCTTCGCTAAGAACGCCTACATGGGTGCTCAGAACATTACGGAGATTGCTGGGATGATTGTCACTGGTAACGTTCGTGCTCTAGGGCATGGTA  >  NC_001604.1/32723‑33322`

  

Alignment Legend

Aligned base mismatch/match (shaded by quality score): `ATCG/ATCG` `< 3 ≤` `ATCG/ATCG` `< 11 ≤` `ATCG/ATCG` `< 24 ≤` `ATCG/ATCG` `< 36 ≤` `ATCG/ATCG` `< 38 ≤` `ATCG/ATCG`

Unaligned base: `atcg`    Masked matching base: `atcg`    Alignment gap: `‑`    Deleted base: `‑`
